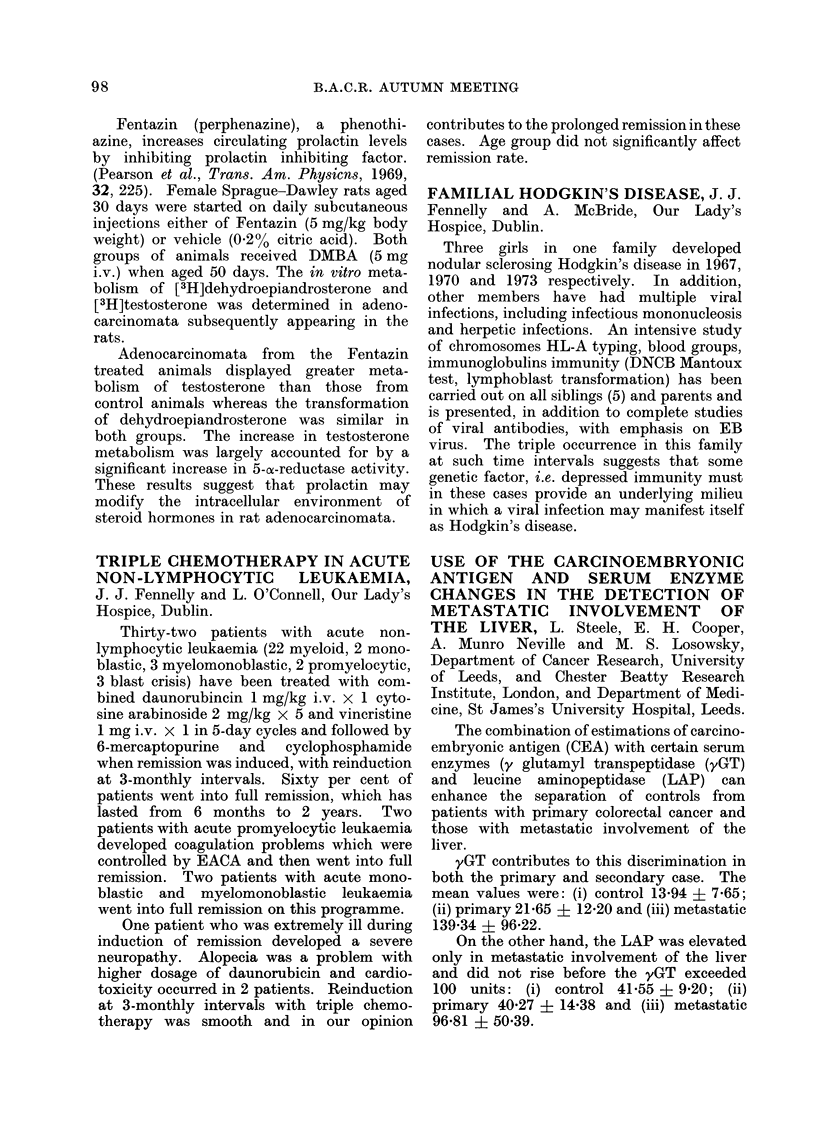# Proceedings: Triple chemotherapy in acute non-lymphocytic leukaemia.

**DOI:** 10.1038/bjc.1974.34

**Published:** 1974-01

**Authors:** J. J. Fennelly, L. O'Connell


**TRIPLE CHEMOTHERAPY IN ACUTE NON-LYMPHOCYTIC LEUKAEMIA,** J. J. Fennelly and L. O'Connell, Our Lady's Hospice, Dublin.

Thirty-two patients with acute non-lymphocytic leukaemia (22 myeloid, 2 monoblastic, 3 myelomonoblastic, 2 promyelocytic, 3 blast crisis) have been treated with combined daunorubincin 1 mg/kg i.v. × 1 cytosine arabinoside 2 mg/kg × 5 and vincristine 1 mg i.v. × 1 in 5-day cycles and followed by 6-mercaptopurine and cyclophosphamide when remission was induced, with reinduction at 3-monthly intervals. Sixty per cent of patients went into full remission, which has lasted from 6 months to 2 years. Two patients with acute promyelocytic leukaemia developed coagulation problems which were controlled by EACA and then went into full remission. Two patients with acute monoblastic and myelomonoblastic leukaemia went into full remission on this programme.

One patient who was extremely ill during induction of remission developed a severe neuropathy. Alopecia was a problem with higher dosage of daunorubicin and cardiotoxicity occurred in 2 patients. Reinduction at 3-monthly intervals with triple chemotherapy was smooth and in our opinion contributes to the prolonged remission in these cases. Age group did not significantly affect remission rate.